# ﻿*Chlorencoelia* (Leotiomycetes, Helotiales, Cenangiaceae) in New Zealand

**DOI:** 10.3897/mycokeys.119.152958

**Published:** 2025-07-22

**Authors:** Katarzyna Patejuk, Peter R. Johnston, Duckchul Park, Mahajabeen Padamsee

**Affiliations:** 1 Department of Plant Protection, Division of Plant Pathology and Mycology, Wrocław University of Environmental and Life Sciences, Wrocław, Poland Wrocław University of Environmental and Life Sciences Wrocław Poland; 2 Manaaki Whenua Landcare Research, Private Bag 92170, Auckland 1072, New Zealand Manaaki Whenua Landcare Research Auckland New Zealand

**Keywords:** Ascomycota, Australasia, ITS phylogeny, Leotiomycetes, light microscopy

## Abstract

*Chlorencoelia*, a genus of saprotrophic, wood-inhabiting fungi, has long been considered to include only a few species with wide geographic distributions. Recent DNA sequencing and morphological analyses of specimens from New Zealand, deposited in the New Zealand Fungarium (PDD), have revealed that all local collections previously identified as *C.torta* or *C.versiformis* represent three distinct species, two of them previously unnamed. These species include *C.olivacea*, originally described from Tasmania, together with the newly named *C.australis* and *C.northlandica*. *Chlorencoeliaaustralis* has been found in both Australia and New Zealand. These species exhibit subtle yet unique morphological characteristics, including differences in ascospore size and shape, lipid body arrangements, the shape of tomentum hyphae, and the colour of refractive vacuolar bodies in the paraphyses and tomentum hyphae. Phylogenetic analyses confirm the divergence of these New Zealand and Australian species from previously described taxa. This study underscores the hidden diversity within *Chlorencoelia* and highlights the importance of integrating genetic and morphological data to refine fungal taxonomy.

## ﻿Introduction

*Chlorencoelia* is a genus of fungi in the family Cenangiaceae, within the class Leotiomycetes. This genus was established by Dixon (1975) and comprises five species of saprotrophic, wood-inhabiting cup fungi found in various regions worldwide (Ren and Zhuang 2014). Dixon (1975) accepted two species in the genus: *C.versiformis*, reported as widespread in north temperate regions, and *C.torta*, also widespread in north temperate regions, as well as in Australasia and tropical America. A third species from India, *C.indica*, was transferred to this genus following taxonomic revisions from its original classification as *Midotisindica* (Zhuang 1988). The description of *C.indica* provided by Sharma and Thind (1983, as *Cordieritesindicus*) mentions an ionomidotic reaction, i.e., exuding a deep violet colour with KOH—a characteristic of Cordieritidaceae but lacking in Cenangiaceae (Jaklitsch et al. 2016)—suggesting that *M.indica* does not belong in *Chlorencoelia*. In the past decade, two additional species have been described: *C.ripakorfii* from South America (Iturriaga and Mardones 2013) and *C.macrospora* from China (Ren and Zhuang 2014).

*Chlorencoeliatorta* was first reported from New Zealand by Dennis (1961, as *Chlorospleniumrugipes*) and accepted as present in New Zealand by Dixon (1975). Dixon (1975) also placed the Australian species *Chlorospleniumrodwayi* (originally named by Rodway (1925) as *Ciboriaolivacea*) in synonymy with *C.torta*. *Chlorencoeliaversiformis* was reported from New Zealand by McKenzie et al. (2000), based on specimens identified as this species in the New Zealand Fungarium / Te Kohinga Hekaheka o Aotearoa (PDD). A genome generated from one of the New Zealand specimens (PDD 99091, culture ICMP 21732) was used to confirm that *Chlorencoelia* belongs to the Cenangiaceae (Johnston et al. 2019, as *C.torta*).

Recent DNA sequencing of *Chlorencoelia* specimens collected from New Zealand forests suggests that they represent three distinct species that had not previously been recognised as distinct morphologically. Here, we use specimens from New Zealand in PDD to compare them both genetically and morphologically with the named species in the genus for which DNA sequence data are available through GenBank®.

## ﻿Methods

### ﻿Fungal collection

Most of the specimens examined in this study are from New Zealand (27 specimens), with two specimens each from Australia and Tasmania. Fifteen of the more recently collected specimens were cultured from germinated ascospores. The nuclear Internal Transcribed Spacer (ITS) region sequences used in this study were generated from these cultures or from apothecial tissue taken directly from recently collected specimens.

Dried specimens have been accessioned into the New Zealand Fungarium (PDD), and living cultures have been deposited in the International Collection of Microorganisms from Plants (ICMP). Details of the specimens examined are listed under the descriptions of the new species.

### ﻿Morphological analysis

Morphological structures are described according to Dixon (1975), with some modifications. Dixon used the undefined terms ‘guttules’ and ‘granules’ to describe the coloured structures within paraphyses and the cells that form a layer on the outside of the receptacle, which he referred to as tomentum hyphae. These coloured structures appear to be equivalent to refractive vacuolar bodies (VBs), as described by Baral (1992), and we use Baral’s term in our descriptions. Dixon also used the term ‘guttules’ to describe differentiated globose structures inside the ascospores. These appear to be equivalent to lipid bodies (LBs) sensu Baral (1992), and we likewise use Baral’s term in our descriptions. The outermost layer of cells of the ectal excipulum was described by Dixon as ‘tomentum hyphae’. In the two species he described, this layer was highly differentiated, with the cells having a hair-like appearance, especially in *C.versiformis*. Although the outer excipular layer of the New Zealand species treated here is often less well differentiated, we retain the term “*tomentum hyphae*” for the outermost layer of excipular cells.

Small pieces of hymenial and excipular tissue were taken from dried specimens, rehydrated in water or 3% KOH, then placed in Melzer’s reagent (MLZ) or in Lugol’s iodine (IKI) for examination of amyloid reactions in the asci, ascospore morphology, and observations of VBs within the paraphyses and outer excipular cells. Apothecia were also rehydrated in 3% KOH, and vertical sections were obtained using a freezing microtome at a thickness of approximately 12 µm; these sections were then mounted in lactic acid.

All available specimens of *Chlorencoelia* deposited in the PDD collection (n = 15) were used for morphological examination. Among them, four specimens were identified as *C.australis*, two as *C.northlandica*, and nine as *C.olivacea*. In addition, one specimen of *C.olivacea* from outside the PDD collection was examined (K(M) 159560, Beaton Victorian Discomycetes No. 113). Morphological structures were measured from all specimens included in the species examination.

Two specimens were available for microscopic examination while still alive (PDD 107569, PDD 124454). Small pieces of hymenial tissue were mounted in water, gently teased apart, and examined immediately.

In addition, one specimen from North America (PDD 26971), morphologically typical of *C.torta* as described by Dixon (1975), was also examined.

### ﻿DNA isolation, amplification, and sequencing

Small amounts of mycelium from cultures or tissue from apothecia were added to 50 µL of extraction solution from the REDExtract-N-Amp Plant PCR Kit (Sigma-Aldrich, USA), and the mixture was incubated at 95 °C for 10 min. The resulting supernatant was then used for PCR. ITS sequences were generated using the primers ITS1F and ITS4 (White et al. 1990; Gardes and Bruns 1993). The DNA sequences have been deposited in GenBank (Table 1).

**Table 1.** List of species, with details of strain/voucher information, the name that we accept, country of origin, culture collection/voucher number, and GenBank accession number (ITS), used for phylogenetic analyses.

Species as accepted	Origin	Voucher	ITS GenBank accession number
*Chlorencoelia* sp.	South Korea, Wonju	KUS-F52256	JN033400
*Chlorencoelia* sp.	China, AnHui	HMAS 287036	OQ534202
*Chlorencoelia* sp.	China, AnHui	HMAS 285422	OQ534201
*Chlorencoelia* sp.	South Korea	KA13-1232	KR673699
*Chlorencoelia* sp.	Chile	MES-2558	MH930348
*Chlorencoelia* sp.	Taiwan	HB 8415	LT158424
*Chlorencoeliaaustralis*	Australia, Victoria, Errinundra NP, Errinundra Saddle, Rainforest Walk	ICMP 25304	PP701695
*Chlorencoeliaaustralis*	New Zealand, Auckland Islands, Adams Island, McLaren Bay, vic. Hut	ICMP 25691	PQ541258
*Chlorencoeliaaustralis*	New Zealand, Stewart Island, Pryse’s Peak Track	ICMP 21732 ex type	MH682234
*Chlorencoeliaaustralis*	New Zealand, Marlborough, Pelorus Bridge Scenic Reserve	ICMP 25618	PQ533031
*Chlorencoeliaolivacea*	New Zealand, Rangitikei, Petersen Road	PDD 106117	OR565293
*Chlorencoeliaolivacea*	New Zealand, Waikato, Steuart Russell Awakino Beech Reserve	ICMP 25721	PQ541256
*Chlorencoeliaolivacea*	New Zealand, Waikato, Steuart Russell Awakino Beech Reserve	ICMP 25720 ex type	PQ541260
*Chlorencoeliaolivacea*	New Zealand, Otago Lakes, Glenorchy, Paradise Road	PDD 106302	MK432802
*Chlorencoeliaolivacea*	New Zealand, Otago Lakes, Glenorchy, Paradise Road, adjacent beech forest	PDD 106239	MK432798
*Chlorencoeliaolivacea*	New Zealand, Auckland, Manukau City, Totara Park	PDD 122841	PQ533030
*Chlorencoeliaolivacea*	New Zealand, Waikato, Rangitoto Station	ICMP 24559	PQ541257
*Chlorencoeliaolivacea*	New Zealand, Taupo, vic. Kiko Road, near corner Mangatera Road	ICMP 24001	PQ541255
*Chlorencoeliaolivacea*	New Zealand, Kaikoura, Mt Lyford, Crystal Lake Track	PDD114302	OR565298
*Chlorencoelianorthlandica*	New Zealand, Northland, Omahuta Forest, Pukekohe Stream Track	ICMP 25719	PQ541259
*Chlorencoelianorthlandica*	New Zealand, Northland, Whangarei, Pukenui Forest	ICMP 21462 ex type	PQ541254
*Chlorencoeliatorta*	USA, Indiana, Porter County, Chesterton, Indiana Dunes State Park	S.D. Russell iNaturalist # 91595985	OM809323
*Chlorencoeliatorta*	USA, Indiana, Porter County, Chesterton, Indiana Dunes State Park	S.D. Russell iNaturalist # 91584233	OM809329
*Chlorencoeliatorta*	USA, Indiana, Putnam County, Greencastle	S.D. Russell iNaturalist # 92192624	OM809252
*Chlorencoeliatorta*	USA, Tennessee	TU:119690	LT158481
*Chlorencoeliatorta*	USA, Tennessee	TU:119681	LT158480
*Chlorencoeliaversiformis*	Estonia	TU:104550	LT158411
*Chlorencoeliaversiformis*	Estonia	TAAM:179803	LT158427
*Chlorencoeliaversiformis*	Canada, Ontario, Rouge National Urban Park	TRTC 175704	PP386638
*Chlorencoeliaversiformis*	USA, Indiana, Hancock County, Greenfield	S.D. Russell iNaturalist # 8552320	OM747569
*Chlorencoeliaversiformis*	USA, Indiana, Hancock County, Greenfield	S.D. Russell iNaturalist # 8552320	MN906214
*Chlorencoeliaversiformis*	Canada, Quebec, Foret Boucher, Aylmer	DAOMC 251598	MH457140
*Chlorencoeliaversiformis*	USA, Tennessee	TU:119720	LT158479
*Chlorencoeliaversiformis*	Canada, Ontario, Cambridge, rare Charitable, Research Reserve, Grand River	BIOUG24046-D05	KT695372
Chlorociboriaaeruginascenssubsp.australis	New Zealand, Taupo, Pureora Forest Park	ICMP 18765	JN943459

### ﻿Phylogenetic analyses

Sequence reads and the final alignment were trimmed and assembled in Geneious Prime v. 10.2.6 (Biomatters Inc.). To resolve the phylogenetic and systematic placement of the obtained strains, the ITS region sequences were aligned with reference sequences of the genus obtained from GenBank (Table 1). Chlorociboriaaeruginascenssubsp.australis was used as an outgroup.

Multiple sequence alignments were performed using the MAFFT algorithm v. 7.490 (Katoh et al. 2005), as implemented in Geneious Prime v. 2022.1.1. The alignment was used as the input file for ModelTest-NG v. 0.2.0 (Darriba et al. 2020) to select the best-fit model of nucleotide substitution. The best-fit substitution model was calculated separately for maximum likelihood (ML) and Bayesian inference (BI) templates and used in the respective analyses, based on the Bayesian information criterion (BIC). For ML-based phylogenetic inference, we used RAxML-NG v. 1.0.3 (Kozlov et al. 2019) in ‘all-in-one’ analysis mode. Branch support was inferred with 1000 bootstrap replicates. For BI, we used MrBayes v. 3.2.6 (Ronquist et al. 2012). One million generations were run, sampling every 100 generations. Four parallel chains—one cold and three heated—were used. A consensus tree was generated after discarding the first 25% of trees as a burn-in period. Average standard deviations of split frequencies dropped below 0.01 at the end of the runs. The final phylogenetic tree was prepared with FigTree v. 1.4.4 (Rambaut 2018).

## ﻿Results

### ﻿Phylogenetic analyses

For the final phylogenetic reconstruction, an alignment containing 35 ITS sequences (477 bp) was used (Table 1). Maximum likelihood (ML) analysis was performed using the TrNef+G4 model. The Bayesian inference (BI) analyses were conducted using two concurrent runs of four chains, run for 165,300 generations. The first 25,000 trees were eliminated as a burn-in phase.

**Figure 1.**
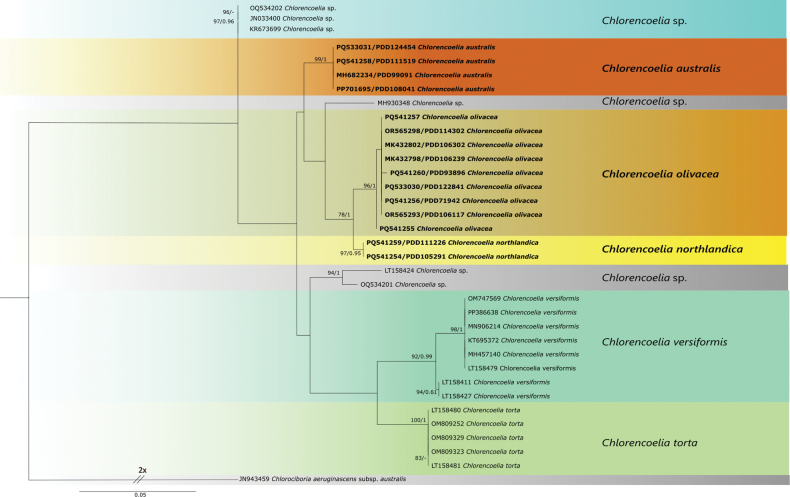
Maximum-likelihood tree inferred from a data set based on rDNA ITS of the sampled *Chlorencoelia* species (species are indicated with coloured boxes, with the names we accept included at the right side of the dendrogram). Newly sequenced specimens of *Chlorencoelia* from New Zealand are indicated in bold. Numbers above branches indicate bootstrap support values (≥75%) and posterior probabilities (PP > 0.90), respectively. The scale bar represents the expected changes per site. Chlorociboriaaeruginascenssubsp.australis was used as an outgroup.

The resulting trees from ML and BI analyses were congruent, showing a well-supported, isolated position of the specimens from New Zealand identified as *C.torta* and *C.versiformis*, as well as other specimens from Asia and southern South America (Fig. 1). The clade accepted here as *C.versiformis* corresponded with *C.versiformis* as accepted by Pärtel et al. (2017). The clade accepted here as *C.torta* includes specimens from North America (close to the species’ type locality) that morphologically match *C.torta* (Pärtel et al. 2017). Specimens from Asia and South America accessioned into GenBank as *C.torta* and *Chlorencoelia* sp. formed three phylogenetically separate groups, assumed to represent additional unnamed species.

The New Zealand species clustered into three well-resolved clades that we recognise here as *C.olivacea*, plus two new species, *C.australis* and *C.northlandica*.

### ﻿Morphological analysis

The guttules and granules described by Dixon (1975) in the paraphyses and tomentum hyphae are visible in water and water + IKI (Lugol’s iodine) but disappear in water + Melzer’s reagent (MLZ) and in KOH. This is characteristic of vacuolar bodies (VBs) sensu Baral (1992), structures found in most Cenangiaceae (Jaklitsch et al. 2016). One departure from the observations of Baral (1992) and Jaklitsch et al. (2016) is that this behaviour is typically lacking in dead tissue. In contrast, with *Chlorencoelia*, these observations were made in both living and dead tissue. In water mounts, the lipid bodies within the ascospores were best observed in MLZ; they often appear to be lacking when spores are mounted in IKI. When mounted in KOH, the lipid bodies within ascospores were not visible in MLZ.

Most of the New Zealand specimens examined from PDD were previously classified as *C.torta* or *C.versiformis*. Our revision revealed distinct morphological characteristics that differentiate the New Zealand specimens from these species, as well as distinguish the three phylogenetically distinct New Zealand species (see Table 2).

**Figure 2.**
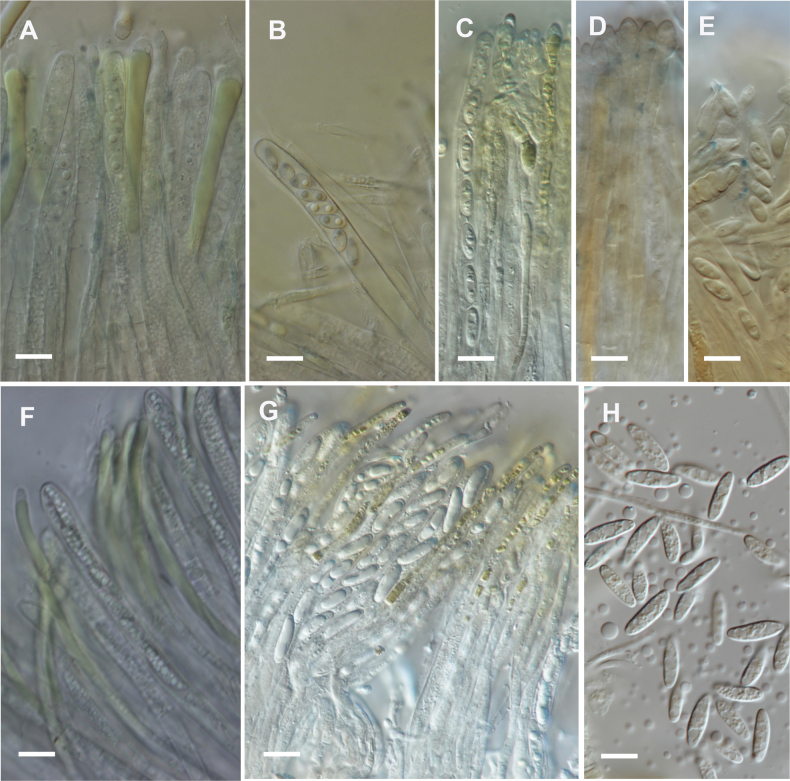
Comparison between living and dead cells of *Chlorencoelia* species. **A–E.***Chlorencoeliaolivacea* (PDD 107569). **A.** Living paraphyses in water; **B.** Living ascospores in water; **C.** Dead, rehydrated paraphyses and ascospores in water +IKI; **D.** Dead, rehydrated paraphyses and ascospores in water + MLZ; **E.** Dead, rehydrated ascospores in water + MLZ; **F–H.***Chlorencoeliaaustralis* (PDD 124454); **F.** Living paraphyses and ascospores in water; **G.** Dead, rehydrated paraphyses and ascospores in water + IKI; **H.** Dead, rehydrated ascospores in water + MLZ. Scale bars = 10 µm.

One specimen of *C.australis* (PDD 124454) and one identified as *C.olivacea* (PDD 107569), based on morphology, were examined in water while still living (Fig. 2). The paraphyses from both specimens formed a single, elongate VB near the apex. In both species, the dried, rehydrated paraphyses had the VBs broken up into many short, rectangular pieces. The lipid bodies in the spores were similar to those in the dried and rehydrated specimens, being biguttulate in *C.olivacea* and multiguttulate in *C.australis*.

Dried apothecia shrunk to about half the size of fresh apothecia (Fig. 4A versus 4C; Fig. 5B versus 5C).

**Figure 3.**
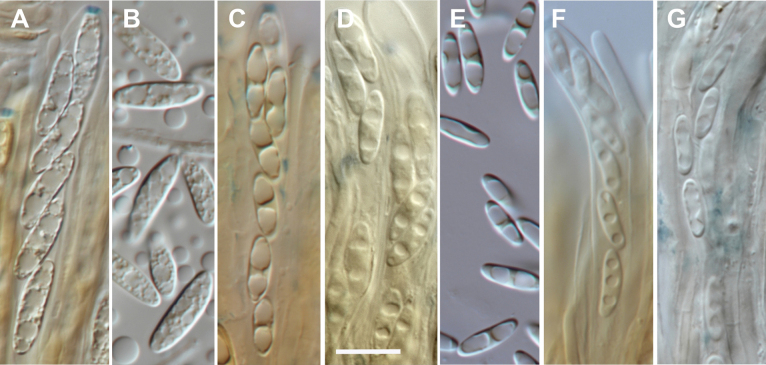
Ascospores from specimens of each of the three New Zealand species, showing variation in size and lipid body arrangement. **A–B.***Chlorencoeliaaustralis*; **C–D.***C.northlandica*; **E–G.***C.olivacea*. **A.**PDD 99091; **B.**PDD 108041; **C.**PDD 111266; **D.**PDD 105291; **E.**PDD 122841; **F.**PDD 71942; **G.**PDD 106302. Scale bar: 10 µm.

The morphological features that distinguish New Zealand’s *Chlorencoelia* species include ascospore size, arrangement of lipid bodies within the ascospores (Fig. 3), and the colour of the VBs in the tomentum hyphae and paraphyses when mounted in water. *Chlorencoeliaolivacea* has the smallest spores, with specimens averaging 8–10.3 × 2.9–3.8 µm, and spores containing 2(–3) large lipid bodies; *C.northlandica* has medium-sized spores, averaging 11.2–11.5 × 3.5–3.8 µm, with spores containing 2(–3) large lipid bodies; *C.australis* has the largest spores, averaging 11.7–15.1 × 3.2–4.3 µm, with spores containing numerous small lipid bodies. The spores in *C.olivacea* were straight or slightly curved, symmetrical at both ends; those in *C.northlandica* were slightly wider towards the apical end; those in *C.australis* were similar in shape at both ends, often slightly curved. Although the intensity of the pigments of the vacuolar bodies in the paraphyses and cells of the tomentum hyphae varied between specimens, it was paler yellow in *C.northlandica* compared to *C.olivacea* and *C.australis*. The cells of the tomentum hyphae were typically short-clavate in all three species, with walls paler than those of the ectal excipulum. The paraphyses of *C.olivacea* tend to be subfusoid, tapering slightly to the rounded apex, whereas those of the other species do not taper.

None of the New Zealand species have the long-cylindric tomentum hyphae characteristic of *C.versiformis* and *C.ripakorfii*. The sometimes dark-green lipid bodies of *C.australis* are similar to those of *C.torta*, but *C.torta* has smaller ascospores with only two lipid bodies.

Cultures grown from germinated ascospores were quite variable in appearance, with few distinguishing features. The 15–30-day-old cultures on Sabouraud Dextrose Agar were sterile, with a diameter of 25–30 mm, and aerial mycelium that was cottony to felted and typically pale yellowish to greenish. Pigmentation in reverse ranged from pale creamy brown to dark greenish brown (often with both pale and dark sectors); however, the *C.northlandica* cultures differed in being almost black in reverse.

**Table 2.** Comparisons of the morphology of the New Zealand *Chlorencoelia* species and species previously reported from New Zealand. The other named *Chlorencoelia* species include *C.macrospora*, with much larger ascospores, 20–40 × 4.5–6 µm (Ren and Zhuang 2014), and *C.ripakorfii*, with long-cylindric, hair-like tomentum hyphae, a species similar to *C.versiformis* but with smaller ascospores (Iturriaga and Mardones 2013).

Species	*C.versiformis*$	*C.torta*#	*C.australis*	*C.northlandica*	*C.olivacea*
**ascus size**	(79–)95–130 (–150) × 5–8 µm	(69–)90–121(–126) × 5–7 µm	90–135 × 9–12 µm	90–127 × 5.5–7 µm	75–104 × 5–9 µm
**spore size**	(10–)11–15 × 2.5–3.5 µm	(5.6–)9–11(–12) × 2–4 (x– 8.5 × 3.2) µm	(9.5–)11–16.5 × (2.5–)3.5–5 (x– 13.5. × 3.6) µm	9.5–13 × 3–4.5 (x– 11.3 × 3.6) µm	8.5–12 × 2.5–3.5 (x– 10.1 × 3.2) µm
**spore shape**	Cylindric-oblong, gently curved	Irregularly ellipsoid, often slightly wider towards the apical end	Oblong-elliptic, gently curved	Oblong-elliptic, flat one side, slightly wider toward apical end	elliptic, symmetrical to each end, sometimes gently curved
**spore lipid bodies**	variable, up to 4 LBs	two LBs	2–3 large LBs plus multiple small LBs	two large LBs	two to three large LBs
**paraphyses**	cylindric, undifferentiated or occasionally subclavate at the rounded apex, VBs yellow to green in water	cylindric, undifferentiated or slightly swollen towards rounded apex, VBs dark green in water	cylindric, VBs bright yellow to green in water	Cylindric or slightly swollen to the rounded apex, VBs pale yellow in water	cylindric, often subfusoid near apex, tapering slightly to rounded apex, VBs bright yellow to green in water
**tomentum hyphae**	25–45 × 5–6 µm, long-cylindric, rarely with green VBs in water	15–25 × 5–10 µm, Subglobose to clavate, containing green or bright yellow VBs in water	15–25 × 8–12 µm clavate, with bright yellow to dark green VBs in water	8–12 × 6–8 µm, globose to subclavate, pale yellow VBs in water	8–17 × 5–10 µm, globose to subclavate, bright yellow or green VBs in water

$ Data from Dixon (1975); # Data from Dixon (1975) and PDD 26971.

### ﻿Taxonomy

#### ﻿ Chlorencoelia australis

Taxon classificationFungiHelotialesHemiphacidiaceae

P.R.Johnst. & Patejuk, sp. nov.

746D6551-F00C-5193-BD85-3F6F31A14E98

MycoBank No: 857863

Fig. 4

##### Etymology.

The species epithet “*australis*,” meaning “southern,” refers to the Southern Hemisphere and Australasian distribution, together with its southern distribution within New Zealand.

##### Type.

New Zealand • Stewart Island: Pryse’s Peak Track, 46.937407S, 168.015158E, on decorticated wood of *Leptospermumscoparium*, coll. P.R. Johnston (D1686), R. Leschen & S. Whitton, 26 Apr 2002 (PDD 99091—**holotype**; ex-type culture ICMP 21732; GenBank ITSMH682234, genome QYAN00000000).

##### Description.

Apothecia 5–10 mm diam., short and broad stipitate, superficial on decorticated and rotting wood, discoid, infundibuliform, gregarious, hymenium steel blue or green when fresh, becoming brown upon drying. Receptacle dark grey-brown, roughened, with a yellow cast when dry. Stipe rugose. In vertical section, ectal excipulum 30–70 μm thick, comprising angular cells 5.5–10 µm diam. oriented nearly perpendicular to the flanks of the apothecium, walls pale brown, outer layer of clavate tomentum hyphae 15–25 × 8–12 μm, walls hyaline, slightly thickened, forming a well-developed layer across the surface of the receptacle, cells containing green to bright yellow vacuolar bodies in water. Medullary excipulum 170–240 μm thick, comprising a loose textura intricata, hyphae 1.5–3.5 µm diam., with walls pale brown, encrusted with dark brown granulations. Subhymenium 35–80 μm high, comprising pale brown, dense textura intricata. Asci 90–135 × 9–12 μm, cylindrical, with tapering stalks, tapering to subtruncate apex, wall thickened at apex, ascus pore blue in IKI and MLZ, croziers at base, 8-spored. Ascospores (9.5–)11–16.5 × (2.5–) 3–4.5 (–5) μm (x– 13.3 × 3.6 µm), elliptical, tapering to rounded ends, slightly curved, 3–4 large, plus several smaller, lipid bodies. Paraphyses 2–2.5 (–3.5) µm diam., undifferentiated to rounded apex, green or bright yellow vacuolar bodies in water.

**Specimens examined.** New Zealand • Taupo: Rangitoto Station, Ranginui Summit, 38.3646°S, 175.474°E, on decorticated wood, coll. P.R. Johnston (D1615), S. Whitton, 16 May 2001 (PDD 99178). • Marlborough: Pelorus Bridge Scenic Reserve, 41.297003°S, 173.572436°E, on dead wood, coll. H.S. Chan, 16 May 2024 (PDD 124454; culture ICMP 25618; GenBank ITSPQ533031). • Buller: Lewis Pass, Nina Valley Track, 42.4623°S, 172.363°E, on *Nothofagus* sp. dead wood, coll. A.E. Bell (AEB496), 11 May 1990 (PDD 73667). • Mid Canterbury: Springfield, Kowai Bush, 43.2893°S, 171.925°E, on rotten wood *Fuscosporasolandri*, coll. J.A. Cooper (JAC 10874), 5 Mar 2009 (PDD 95342). • Dunedin: Swampy Spur Track, 45.8013°S, 170.488°E, on dead wood, coll. D.P. Mahoney (AEB1052), 12 May 2008 (PDD 94231). • Fiordland: Fiordland National Park, Lake Monowai, 45.824°S, 167.433°E, on dead wood of Nothofagaceae, coll. P.R. Johnston (D641), 19 Mar 1991 (PDD 58554); • Hollyford Valley, Lake Marian Track, 44.8003°S, 168.093°E, on dead wood, coll. P.R. Johnston & P.F. Cannon, 14 Feb 2003 (PDD 119575). • Auckland Islands: Adams Island, McLaren Bay, vic. Hut, 50.8667°S, 166.018°E, on rotting wood, coll. P.R. Johnston (AK 1049), 23 Mar 2006 (PDD 111519; culture ICMP 25691; GenBank ITSPQ541258).

Australia • Victoria: Errinundra National Park, Errinundra Saddle, Rainforest Walk, on decorticated wood, coll. P.R. Johnston (AU96-120), 24 May 1996 (PDD 108041; culture ICMP 25304; GenBank ITSPP701695).

##### Notes.

*Chlorencoeliaaustralis* can be distinguished from the other New Zealand species by its larger ascospores, which contain multiple lipid bodies. The layer of tomentum hyphae is better differentiated than the other two species. *C.australis* is also found in Australia. All but one of the New Zealand specimens examined were found in the South Island.

**Figure 4.**
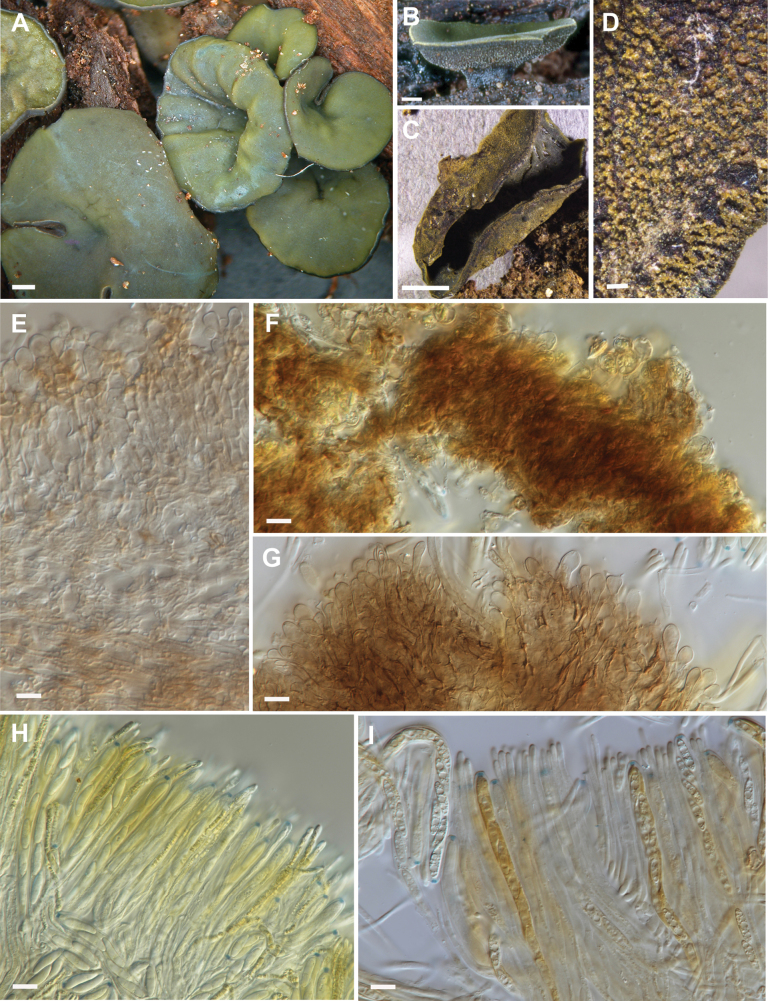
*Chlorencoeliaaustralis*. **A, B.** Fresh specimens, showing green hymenium and dark-coloured receptacle; **C.** Dried specimen; **D.** Detail of underside of dried receptacle, showing yellow bloom; **E.** Excipulum in vertical section, tomentum hyphae to the top; **F.** Squash mount with tomentum hyphae (water + IKI); **G.** Squash mount with tomentum hyphae (water + MLZ); **H.** Paraphyses with yellow vacuolar bodies (water + IKI); **I.** Hymenium with asci, ascospores with lipid bodies, paraphyses (water + MLZ). **A.**PDD 95342; **B.**PDD 58554; **C–D.**PDD 124454; **E.**PDD 111519; **F.**PDD 108041; **G–I.**PDD 99091. Scale bars: 1 mm (**A–C**); 0.1 mm (**D**); 10 µm (**E–I**).

#### ﻿ Chlorencoelia northlandica

Taxon classificationFungiHelotialesHemiphacidiaceae

P.R.Johnst. & Patejuk, sp. nov.

3E2024D3-3A8B-5281-97D4-344DD1AB4F8F

MycoBank No: 857864

Fig. 5

##### Etymology.

The species epithet “northlandica” refers to the Northland region of New Zealand, the only region where this species has been found.

##### Type.

New Zealand • Northland: Whangarei, Pukenui Forest, 35.717566°S, 174.263404°E, on decorticated wood, coll. P.R. Johnston, 22 May 1992 (PDD 105291—**holotype**; ex-type culture ICMP 21462; GenBank ITSPQ541254).

##### Description.

Apothecia 5–10 mm diam., short and broad stipitate, superficial on decorticated wood, discoid, gregarious, hymenium dark green to grey-blue when fresh, becoming brown upon drying. Receptacle dark grey-brown, coarsely roughened, with a dull yellow cast when dry. Stipe rugose. In vertical section ectal excipulum 30–90 μm thick, comprising angular cells 6–8 µm diam. oriented more or less perpendicular to surface of receptacle, cell walls pale brown, a barely differentiated outer layer of tomentum hyphae, cells 8–12 × 6–8 μm, globose to subclavate, walls hyaline, slightly thickened, cells containing pale yellow vacuolar bodies in water. Medullary excipulum 55–340 μm thick comprising loose textura intricata with hyphae 2–3 µm diam., cell walls pale brown, encrusted with scattered dark brown granulations. Subhymenium 30–48 μm high, pale brown, dense textura intricata. Asci 90–127 × 5.5–7 μm, cylindrical with tapering stalks, tapering slightly to rounded apex, wall thickened at apex, ascus pore blue in MLZ and IKI, croziers at base, 8-spored. Ascospores 9.5–13 × 3–4.5 µm (x– 11.3 × 3.6 µm), elliptical, slightly wider towards the upper end, never curved, with 2–3 large lipid bodies. Paraphyses 2.5–3.5 (–4) µm diam., undifferentiated or subclavate at the rounded apex, pale yellow vacuolar bodies in water.

##### Specimen examined.

New Zealand • Northland: Omahuta Forest, Pukekohe Stream Track, 35.2412°S, 173.629°E, on decorticated wood, coll. A. Chinn, 9 May 2017 (PDD 111226; culture ICMP 25719; GenBank ITSPQ541259).

##### Notes.

*Chlorencoelianorthlandica* is known from only two specimens, both of which were collected in Northland. Compared to the other New Zealand species, it has intermediate-sized ascospores. The spores are not curved but often slightly wider toward the upper half of the spore. The vacuolar bodies in the paraphyses and cells of the tomentum hyphae are paler than those of the other two species, where the VBs are bright yellow to dark green.

**Figure 5.**
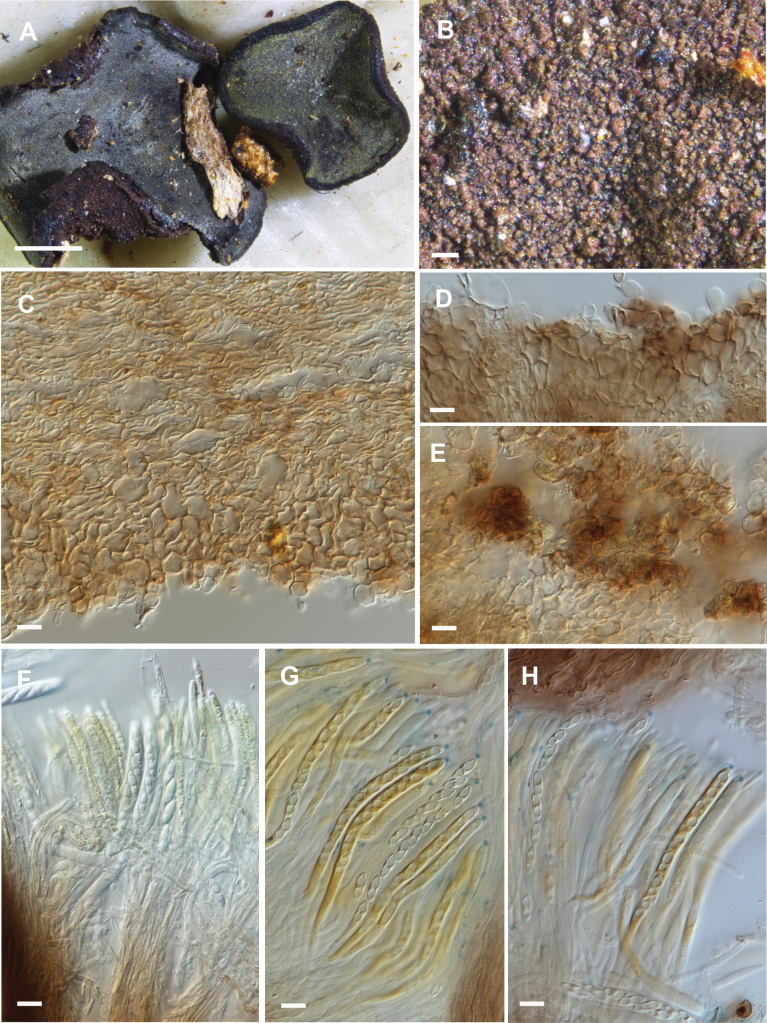
*Chlorencoelianorthlandica*. **A.** Dried specimen with a dark greenish-black hymenium; **B.** Dried specimen, detail of underside of receptacle; **C.** Ascoma in vertical section showing part of medullary excipulum, ectal excipulum, and poorly differentiated tomentum hyphae; **D.** Tomentum hyphae (squash mount, 3% KOH). **E.** Tomentum hyphae with pale yellow vacuolar bodies (squash mount, water). **F.** Paraphyses with pale vacuolar bodies (water + IKI). **G–H.** Ascospores and paraphyses (water + MLZ). **A–C.**PDD 105291; **D–H.**PDD 111226. Scale bars: 1 mm (**A**); 0.1 mm (**B**); 10 µm (**C–H**).

#### ﻿ Chlorencoelia olivacea

Taxon classificationFungiHelotialesHemiphacidiaceae

(Rodway) P.R.Johnst. & Patejuk, comb. nov.

864B37CC-9705-5A58-9848-BE1FEAED3515

MycoBank No: 857865

Fig. 6

≡ Ciboriaolivacea Rodway, Papers and Proceedings of the Royal Society of Tasmania 1924: 105 (1925).
≡ Chlorociboriaversiformisvar.olivacea (Rodway) Dennis, Kew Bull. 13: 340 (1958).
≡ Chlorospleniumrodwayi Korf, Bulletin of the National Science Museum Tokyo 4: 391 (1959).
≡ Chlorospleniumversiformevar.olivacea (Rodway) G.W. Beaton & Weste, Victorian Naturalist 96: 248 (1979).

##### Description.

Apothecia up to 10.5 mm diam., short and broad stipitate, superficial, discoid to infundibuliform, gregarious, hymenium olive green to dark blue-green when fresh, becoming brown to black upon drying. Receptacle brown to dark brown, coarsely roughened, sometimes with a yellow cast when dry. Stipe rugose, blackish. In vertical section ectal excipulum 47–64 μm thick, comprising angular cells 8–10 µm diam., oriented nearly perpendicular to the flanks of the apothecium, walls pale brown, outer layer of globose to subclavate tomentum hyphae 8–17 × 5–10 μm, walls hyaline, slightly thickened, cells containing bright yellow vacuolar bodies in water. Medullary excipulum 60–400 μm thick, comprising hyaline to pale brown loose to compacted textura intricata, hyphae 1.5–2.5 µm diam., cell walls encrusted with dark brown granulations. Subhymenium 24–50 μm high, comprising pale brown, dense textura intricata. Asci 75–104 × 5–9 μm, cylindrical, long tapering basal stalks, flattened across thickened apex, apical pore faint to vivid blue in MLZ and IKI, croziers at base, 8-spored. Ascospores (7–) 8–10.5 (–12) × 2–3.5 (–4) μm (x– 9.5 × 3.2 µm), oblong-elliptical, often gently curved, symmetrical, ends broadly rounded, with 2 (–3) large lipid bodies. Paraphyses 2.5–5 µm diam., cylindric to subfusoid, sometimes tapering towards rounded apex, bright yellow to dark green vacuolar bodies in water.

##### Specimens examined.

New Zealand • Otago Lakes: beech forest beside Glenorchy-Routeburn Road, 44.7824°S, 168.3551°E, on rotting wood of *Fuscosporafusca*, coll. J.A. Cooper (JAC14068), 8 May 2016 (PDD 106239 GenBank sequences: ITSMK432798). • Auckland: Manukau City, Wairere Road, Totara Park, 37.0039°S, 174.913°E, on rotting wood, collector, C. Shirley (CS AK451), 1 Jun 2024 (PDD 122841; GenBank ITSPQ533030). • Waikato: Awakino, Steuart Russell Awakino Beech Reserve, 38.6087°S, 174.669°E on decorticated wood *Fuscosporafusca*, coll. P.R. Johnston (D1539), 25 May 2000 (PDD 71942; culture ICMP 25721; GenBank ITSPQ541256); • Awakino, Steuart Russell Awakino Beech Reserve, 38.576667°S, 174.677627°E, decaying wood of *Fuscosporafusca*, coll. B.C. Paulus and A.J. O’Donnell (AOD 161), 16 Apr 2007 (PDD 93896; culture ICMP 25720; GenBank ITSPQ541260). • Bay of Plenty: Athenree Forest, 37.462°S, 175.915°E, on bark of fallen rotten wood, coll. P.K.C. Austwick, 8 May 2003 (PDD 78394). • King Country: Rangitoto Station, 38.3397°S, 175.4376°E, on decorticated wood, coll. P.R. Johnston (D2050), B.C. Paulus, A. O’Donnell, 14 Apr 2007 (culture ICMP 24559; GenBank ITSPQ541257). • Taupo: vic. Kiko Rd, 38.8997°S, 176.122°E, on Nothofagaceae sp. decorticated wood, coll. P.R. Johnston (D1605), S. Whitton, 4 May 2001 (culture ICMP 24001, GenBank ITSPQ541255); • Ohakune, Rangataua Forest & Ecological area, 39.0123°S, 175.661°E, on dead wood *Fuscosporafusca*, coll. D.P. Mahoney (AEB909), 5 Apr 2005 (PDD 83070). • Rangitikei: Petersen Road, 39.959671°S, 176.010537°E, on wood of *Fuscosporafusca*, coll. J.A. Cooper (JAC 13914), 18 May 2015 (PDD 106117; GenBank ITSOR565293). • Kaikoura: Mt Lyford, Crystal Lake Track, 42.478256°S, 173.148937°E, on wood of *Fuscosporacliffortioides*, coll. J.A. Cooper (JAC 17192), 29 Mar 2022 (PDD 114302; GenBank ITSOR565298); • Mt Lyford, Crystal Lake Track, 42.4783°S, 173.1489°E, on dead wood *Fuscosporacliffortioides*, coll. J.A. Cooper (JAC 17192), 29 Mar 2022 (PDD 114302; GenBank ITSOR565298). • Westland: Haast Pass Summit, 44.1093°S, 169.352°E, on fallen wood, 16^th^ Fungal Foray of New Zealand, 9 May 2002 (PDD 75507). • Otago Lakes: Glenorchy, Paradise Road, 44.848043°S, 168.346777°E, on rotting stump of *Fuscosporafusca*, coll. J.A. Cooper (JAC 14135), 8 May 2016 (PDD 106302; GenBank ITSMK432802); • Glenorchy, Rees Road, Invincible Creek, 44.731°S, 168.456°E, on decorticated wood, coll. P.R. Johnston (D2409), 7 May 2016 (PDD 107569). Fiordland: Te Anau, Kepler Track, Iris Burn Valley, on dead wood, coll. T. Atkinson, 12 Mar 2003 (PDD 78392). • Stewart Island: on dead wood *Pterophyllaracemosa*, coll. J.M. Dingley, 18 Feb 1954 (PDD 19026).

Australia • Victoria: Lake Mountain Rd, Vic. Marysville, coll. G.A. Crichton, 24 Mar 1963 (K(M) 159560, Beaton Victorian Discomycetes No. 113).

##### Notes.

*Chlorencoeliaolivacea* has smaller ascospores than the other species treated in this study. The spores are often slightly curved and have two large lipid bodies. *Chlorencoelianorthlandica* has only slightly larger ascospores but also differs in having paler VBs in the paraphyses and tomentum hyphae, as well as less well-differentiated tomentum hyphae.

Originally described from Tasmania, this species has also been found in Victoria in eastern Australia. Its occurrence in New Zealand is based on the morphological similarity of the New Zealand specimens to an Australian specimen from Victoria (K(M) 159560) and to the description of the type specimen provided by Dennis (1958) as Chlorociboriaversiformisvar.olivacea.

*Chlorociboriaolivacea* is the most common species in New Zealand, found on decaying wood throughout the country, and many of the specimens are collected from the wood of *Nothofagus* species.

**Figure 6.**
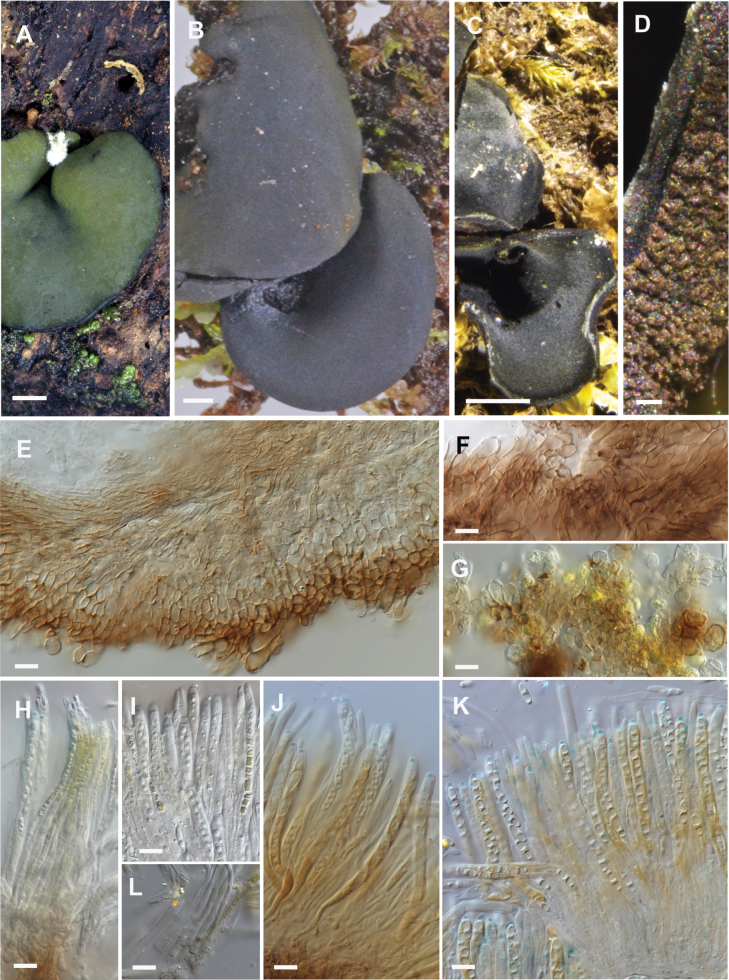
*Chlorencoeliaolivacea*; **A, B.** Fresh specimen, hymenium dark green or bluish; **C.** Dried specimen, black hymenium; **D.** Dried specimen, detail of underside of receptacle; **E.** Ascoma in vertical section; **F.** Detail of tomentum hyphae showing clavate shape (3% KOH). **G.** Detail of tomentum hyphae showing yellow VBs (water). **H.** Paraphyses with yellow VBs, asci with blue pore (water + IKI). **I.** Paraphyses with yellow VBs and tapering toward a rounded apex, asci lacking blue pore (water + IKI). **J.** Ascospores with 2–3 LBs (water + MLZ). **K.** Ascospores with two large LBs (water + MLZ). **L.** Croziers at ascus base (water). **A.**PDD 106117; **B–C.**PDD 106302; **D, I, J.**PDD 71942; **E, G.**PDD 93896; **F.**PDD 106177; **H, K, L.**PDD 122841. Scale bars: 1 mm (**A–C**); 0.1 mm (**D**); 10 µm (**E–K**).

## ﻿Discussion

Our studies indicate that all specimens of *Chlorencoelia* collected from New Zealand represent previously unrecognised diversity that exhibits clear morphological and genetic distinctions from currently recognised species of the genus. None of the specimens studied correspond to *C.torta* or *C.versiformis*, previously considered part of New Zealand’s mycobiota. This pattern is familiar. As the Helotiales of New Zealand become genetically characterised, it is common to find that species reported from New Zealand that were initially named from north temperate regions and previously thought to have very wide geographic distributions are shown to be genetically distinct. In many cases, the unique New Zealand taxa remain unnamed. Examples amongst Leotiomycetes include *Chlorosplenium* (Stallman et al. 2024), *Chlorociboria* (Johnston and Park 2005), *Hyphodiscus*, a genus with at least six unnamed species (Johnston 2022), and *Marthamyces* (Johnston and Park 2019), a paper that provides a new name for a very common *Metrosideros*-inhabiting fungus that had previously been incorrectly identified as the *Eucalyptus*-inhabiting *M.emarginatus*.

Two of the species, *Chlorencoeliaaustralis* and *C.olivacea*, are also known from Australia. The occurrence of *C.australis* in the two countries is supported by matching DNA sequences, while *C.olivacea* is accepted for New Zealand on the basis of matching morphology; this distribution is still to be validated genetically.

Most *Chlorencoelia* species are difficult to distinguish morphologically. The genetic data presented here allowed for the recognition of subtle differences in features such as ascospore size and shape, the arrangement of ascospore lipid bodies, the shape of tomentum hyphae, and the colour of the refractive vacuolar bodies in the paraphyses and tomentum hyphae as taxonomically significant. Our study highlights the previously unrecognised diversity of *Chlorencoelia*, which has long been limited to five species. Molecular analyses indicate that there are sequences in GenBank, variously referred to as *Chlorencoelia* sp. and *Chlorencoeliatorta*, that represent additional unnamed species in other parts of the world.

## Supplementary Material

XML Treatment for Chlorencoelia australis

XML Treatment for Chlorencoelia northlandica

XML Treatment for Chlorencoelia olivacea

## ﻿Additional information

### Use of AI

No use of AI was reported.

### Author ORCIDs

Katarzyna Patejuk https://orcid.org/0000-0001-7236-8005

Peter R. Johnston https://orcid.org/0000-0003-0761-9116

Duckchul Park https://orcid.org/0000-0002-2671-180X

Mahajabeen Padamsee https://orcid.org/0000-0002-0741-3014

### Data availability

Dried specimens (holotype, isotype, and other specimens) are stored in the fungal collection of the New Zealand Fungarium/Te Kohinga Hekaheka o Aotearoa, Auckland, New Zealand. Fungal cultures are stored in the publicly available International Collection of Microorganisms from Plants/Te Kohinga Hekaheka Ora o Aotearoa, Auckland, New Zealand. Sequences obtained in the present study have been deposited in the GenBank NCBI nucleotide sequence database (www.ncbi.nlm.nih.gov/genbank).
